# Height and Risk of Vitiligo: A Nationwide Cohort Study

**DOI:** 10.3390/jcm10173958

**Published:** 2021-08-31

**Authors:** Young-Bok Lee, Hei-Sung Kim

**Affiliations:** 1 Department of Dermatology, Uijeongbu St. Mary’s Hospital, The Catholic University of Korea, Seoul 06591, Korea; lyb80@catholic.ac.kr; 2 Department of Dermatology, Incheon St. Mary’s Hospital, The Catholic University of Korea, Seoul 06591, Korea

**Keywords:** height, risk, vitiligo, nationwide cohort study

## Abstract

Adult height is linked to the risk of several diseases, but its association with vitiligo has not been established. This study aimed to investigate the relationship between adult height and vitiligo incidence. Korean nationwide claims data from 15,980,754 individuals (20 years of age or older) who received a health checkup during the period 2005–2008, were examined. Subjects were categorized into age- and gender-specific height quintiles. Participants were followed until vitiligo diagnosis or until the end of 2015. The Cox proportional-hazards model for cumulative risk was computed for height categories. During the follow-up period, 29,196 cases (136,020,214 person-years) of newly diagnosed vitiligo were reported. A positive association was found between height and risk of vitiligo in which the hazard ratio between the highest and lowest quintiles of height was 1.36 (95% confidence interval: 1.31–1.42). While more diverse cohort studies are needed, our findings suggest that taller stature increases the risk of vitiligo.

## 1. Introduction

Vitiligo is an acquired skin condition typified by white patches resulting from selective loss of melanocytes [1,2,3]. With an estimated global prevalence of 1% [4,5,6], vitiligo poses a major challenge to both patients and societies worldwide [7]. Particularly frustrating are the unpredictable disease course and lack of FDA-approved effective therapy [8,9]. Despite the effort expended to develop ground-breaking therapeutics, vitiligo remains incurable [10]. Identification of risk factors that enable early screening of susceptible individuals would be beneficial.

Vitiligo is believed to have a complex pathogenesis involving both genetics and environmental triggers [2]. Estimates of vitiligo heritability range from 0.5 to 0.8 [11,12], and findings of increased frequency of vitiligo among first-degree relatives and a strong spousal concordance [13,14] imply substantial genetic influence on vitiligo risk. As for the environmental insult, the Koebner phenomenon is prevalent in vitiligo patients [15], which suggests that skin damage is an important provocation factor [16].

Interestingly, body height is determined by interactions among genetic predisposition and environmental factors (i.e., nutrition and sleep) during childhood and adolescence [17,18] and can serve as a disease indicator [19]. Adult height has been linked with a number of diseases. Taller stature was reported to increase the risk of atrial fibrillation [20,21,22], venous thromboembolism [21,22], meningioma [23], vasculitis [22], actinic keratosis [24], and cancers including melanoma [22,25] and non-melanoma skin cancer [26,27].

Unlike the aforementioned diseases, the relation between body height and vitiligo has not been investigated. In this study, we assessed the association between adult height and risk of vitiligo in South Korea, an ethnically homogeneous nation, using a nationwide dataset.

## 2. Materials and Methods

### 2.1. Data Source

South Korea utilizes a mandatory National Health Insurance (NHI) system where members are obligated to undergo biennial health screening from age 40. We retrieved data from two NHI databases, the Health Examination database and the NHI service claims database. The first dataset was used to select the subjects and collect data on height and possible confounding factors. The NHI claims dataset was analyzed to detect vitiligo occurrence in selected individuals. The diagnostic information in the NHI claims database is documented based on the International Classification of Disease (ICD)-10 code.

### 2.2. Ethics

The Ethics Committee of Incheon St. Mary’s Hospital, The Catholic University of Korea, reviewed and accepted the study protocol (OC17ZESI0052). We gained permission from the Korea Disease Control and Prevention Agency to retrieve information from the NHI database.

### 2.3. Study Subjects

We recruited subjects who were older than 20 and had undergone a health checkup between 2005 and 2008. In cases in which subjects received multiple health screenings during the time period, data from the first health exam (index date) were used. Individuals with any missing data were excluded from the analysis. The cohort was followed on the NHI claims database until vitiligo diagnosis (ICD-10 code, L80) or until December 31, 2015. A total of 15,980,754 subjects were ultimately included in the study cohort after excluding those with pre-existing vitiligo.

### 2.4. Data Collection and Definitions of Comorbidities and Other Variables

The health exam involves a survey and direct measurements. Information on age, gender, income level (dichotomized at the lowest 20%), alcohol consumption, and smoking history was collected through a questionnaire. Anthropometric measurements (i.e., height and weight) were performed using a standard scale with individuals wearing light clothing and no shoes. The body mass index (BMI) was calculated as weight (kg) divided by height squared (m^2^). Blood pressure (BP) was measured routinely while sitting after a five-minute rest time. Blood was sampled after an eight-hour overnight fast to measure blood glucose and total cholesterol.

To remove the confounding effect of comorbid diseases, we identified cases of diabetes mellitus (DM), hypertension, and dyslipidemia. DM was defined based on the use of insulin or an oral hypoglycemic agent (ICD-10, E10-14) or a fasting glucose level ≥7 mmol/L. Hypertension was defined as the use of an antihypertensive agent (ICD-10, I10-15) or systolic/diastolic BP ≥ 140/90 mmHg. Dyslipidemia was defined as the use of a lipid-lowering agent (ICD-10, E78) or total cholesterol ≥ 6.21 mmol/L. Both the NHI claims database and the Health Exam database were used to identify the presence of a comorbidity.

### 2.5. Statistical Analysis

Descriptive statistics are shown as mean ± standard deviation or percentage. Subjects were classified into age- and gender-specific quintiles based on index height (Supplementary Table S1). A Cox proportional hazards model was adopted to determine the independent effect of height on vitiligo development after controlling for age and gender in model 1. Age, gender, weight, income, alcohol consumption, smoking history, presence of hypertension, presence of DM, and presence of dyslipidemia were controlled for in model 2. The hazard ratio (HR) and 95% confidence interval (CI) for each height quintile relative to the lowest quintile were calculated. Subgroup analyses were performed based on gender and age (>65 years and ≤65 years). The proportional hazard assumptions were validated with the log–log cumulative survival graph and the time-varying factor Cox model. We used the SAS software ver. 9.4 (SAS Institute, Cary, NC, USA) for all analyses where *p* ≤ 0.05 was recognized as statistically significant.

## 3. Results

### 3.1. Characteristics of the Study Cohort

Our cohort consisted of 15,980,754 participants who were grouped into gender- and age-adjusted height quintiles. The baseline (index date) characteristics of the study cohort are presented in Table 1.

### 3.2. Vitiligo Risk Stratified by Height

**Figure 1:**
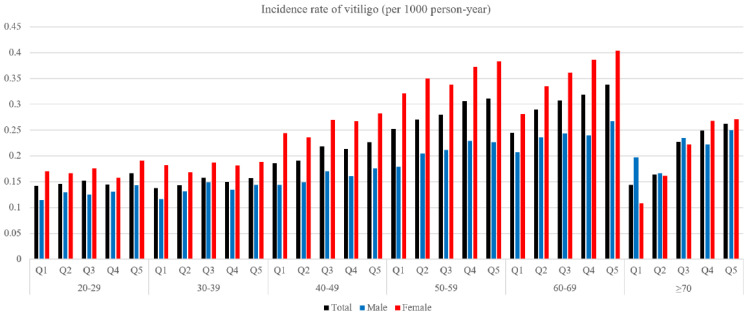
The unadjusted incidence rate of vitiligo (per 1000 person-year) according to index age, height, and gender.

**Figure 2:**
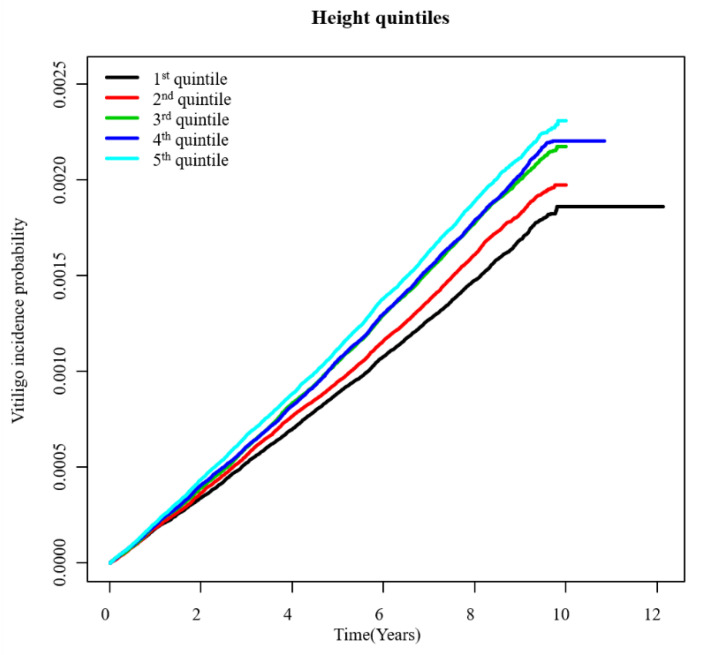
Adjusted cumulative incidence of vitiligo by height (Q1–Q5).

The total number of newly diagnosed vitiligo cases was 29,196 during a follow-up period of 136,020,214 person-years (Table 2). Figure 1 presents an unadjusted incidence rate (per 1000 person-years) of vitiligo by index age (10-year age ranges), height (Q1–Q5), and gender. The cumulative incidence of vitiligo for each height quintile after covariate (i.e., age, gender, BMI, presence of hypertension, presence of diabetes, presence of dyslipidemia, smoking, alcohol consumption, and income status) adjustment is shown in Figure 2. With the Q1 (shortest) group as reference, the hazard ratio (HR) and 95% confidence interval (CI) of the Q5 (tallest) group was 1.36 (95% CI, 1.31–1.41) under the multivariable model (model 2) (Table 2).

#### 3.2.1. Subgroup Analysis by Gender

Among the male cohort, 12,396 subjects were newly diagnosed with vitiligo during a follow-up period of 72,959,513 person-years. Vitiligo incidence (per 1000 person-years) of the Q5 quintile was 0.18. With the Q1 group as reference, the HR and 95% CI of the Q5 group were 1.36 (95% CI, 1.28–1.45) under model 2 (Table 2).

Of the total, 16,800 female subjects were diagnosed with vitiligo during the observation period (63,060,700 person-years). Vitiligo incidence of the Q5 group was 0.29 per 1000 person-years. The HR and 95% CI of the Q5 group were 1.35 (1.28–1.42) under multivariate analysis (model 2) (Table 2).

#### 3.2.2. Subgroup Analysis by Age

In a recent epidemiological study (data not shown) of Koreans, vitiligo was shown to have two prevalence peaks (in the first decade (0–9 years) and the fifth decade (60–69 years)). Accordingly, we chose 65 as the cutoff value for our two subgroups (“65 years and higher” versus “under 65 years”). Upon subgroup analysis by age, patients 65 and higher showed a vitiligo incidence of 0.31 (Q5). The HR and 95% CI of the Q5 quintile were 1.44 (1.32–1.56) under the multivariable model (model 2) (Table 2).

The Q5 group of individuals under 65 presented with a vitiligo incidence of 0.22 per 1000 person-years. Using the Q1 group as reference, the model 2 HR and 95% CI of the Q5 group were 1.37 (95% CI, 1.31–1.44) (Table 2).

## 4. Discussion

Findings from our nationwide cohort study suggest that adult height positively correlates with risk of vitiligo in Koreans. The association was stronger in the elderly population (age ≥ 65 versus age < 65). To the best of our knowledge, this is the first study to analyze the relationship between body height and incidence of vitiligo.

Epidemiological findings including nationwide population studies from Korea have shown strong associations between vitiligo and autoimmune diseases (systemic lupus erythematosus, alopecia areata, thyroiditis, type 1 diabetes, and rheumatoid arthritis) [28,29,30,31,32,33], which corroborates the autoimmune nature of vitiligo [2]. Interestingly, male body height has been suggested as a cue for immune efficacy [34]. Greater height-growth velocity was involved with islet autoimmunity and type 1 diabetes [35], linking tall stature to autoimmunity and ultimately to vitiligo.

Insulin-like growth factor (IGF) signaling is involved in growth and metabolic processes [35]. IGF-1 encourages longitudinal bone growth [36], and both IGF-1 and IGF-2 take part in cancer progression [37]. Vitiligo melanocytes produce insulin-like growth factor-binding protein (IGFBP) 3 [38], the expression of which is, like IGF-1, growth hormone-dependent [39]. In addition, metformin, which regulates IGF level, has been shown to modulate height [40], inhibit tumor growth [41,42], and lower the risk of incident vitiligo [43]. This connects vitiligo to height and cancer incidence (which is known to be related to tall stature) [44].

Genetic factors contribute strongly to adult height, and certain genes linked with height [22] are involved in vitiligo-regulatory pathways. These include JNK (jun amino terminal kinase), PI3K (phosphatidylinositol 3 kinase), JAK1 (Janus kinase 1), CREB (cyclic AMP responsive element-binding) protein, ERK (extracellular signal-regulated kinase), and mTOR (mammalian target of rapamycin) [45,46,47,48]. JNK is involved in growth plate development and chondrocyte differentiation [49] and suppresses melanogenesis by interfering with CREB-regulated transcription coactivator 3-dependent microphthalmia-associated transcription factor (MITF) activation [50]. PI3K/Akt signaling is a key regulator in terminal chondrocyte differentiation [51] as well as in cell proliferation and apoptosis of melanocytes [52]. The JAK/STAT (signal transducer and activator of transcription) pathway constitutes the principal signaling pathway for growth factor receptors [53] which affect body height. Interestingly, JAK inhibitors have recently been explored as a promising novel treatment option for vitiligo by inducing re-pigmentation [54]. ERK inhibits chondrocyte differentiation, and its hyperactivation contributes to short stature [55]. At the same time, the ERK/CREB pathway is known to enhance melanin synthesis via upregulation of MITF and TRP-1 [56]. The activity of mTOR is increased by mitogenic signals through PI3K/AKT [57] and contributes to chondrocyte hypertrophy and differentiation [58]. The mTOR pathway is also involved in melanocyte survival in response to UV radiation and oxidative stress [59] and its modulation is thought to offer better approaches for the clinical management of vitiligo [60].

Environmental stress can trigger the onset of vitiligo [10,11], especially in adulthood. Striae-induced Koebner phenomenon is observed in vitiligo [61,62,63], and the fact that striae are more common in taller individuals with intense growth spurts can explain the increased risk of vitiligo with height.

Accumulative UV-radiation exposure is another known environmental trigger of vitiligo [11]. UV irradiance increases with altitude, which also applies to body height, thus contributing to the higher incidence of vitiligo as well as skin cancer [44] and actinic keratosis [24] in taller individuals. This is especially true in the elderly population who have high cumulative exposure to UV radiation.

Strengths of our cohort study include its large population derived from a nationwide database. This is a quality controlled NHI claims database with a sample size of 50 million [64]. This provided us with the ability to control for potential confounders such as age, gender, BMI, presence of hypertension, presence of DM, presence of hyperlipidemia, smoking, alcohol consumption, and income status. The homogeneity of the Korean population also adds strength to our findings.

The limitations of our study are a relatively short follow-up period and the lack of information on vitiligo severity, family history, use of medications, and occupational information related to UV-radiation exposure. Also, since the study cohort was recruited from health checkup examinees, the study was not free from selection bias.

In conclusion, adult body height was significantly linked to an increased risk of vitiligo in this nationwide, prospective cohort study. Further research on the mechanisms that underlie the association between height and vitiligo could assist in disease prevention.

## Figures and Tables

**Table 1:** Characteristics of the study cohort at the index date.

Height ^a^					
	Q1 (*N* = 3,093,715)	Q2 (*N* = 3,179,095)	Q3 (*N* = 3,442,765)	Q4 (*N* = 3,007,857)	Q5 (*N* = 3,179,095)
Age (year) ^b^	46.9 ± 14.9	46.5 ± 14.7	46.5 ± 14.5	46.2 ± 14.3	45.8 ± 14.4
Male Gender	1,692,262 (54.7)	1,745,323 (54.9)	1,752,367 (50.9)	1,555,062 (51.7)	1,656,308 (52.1)
Weight ^b^	57.7 ± 9.7	61.3 ± 10.2	63.2 ± 10.6	65.2 ± 11.1	69.2 ± 12.2
BMI (kg/cm^2^) ^b^	23.7 ± 3.2	23.6 ± 3.2	23.6 ± 3.2	23.6 ± 3.2	23.5 ± 3.2
<18.5	113,930 (3.7)	120,244 (3.8)	133,139 (3.9)	127,272 (4.2)	142,331 (4.4)
18.5–23	1,212,028 (39.2)	1,248,787 (39.3)	1,364,068 (39.6)	1,212,699 (40.3)	1,315,805 (40.4)
23–25	757,897 (24.5)	791,295 (24.9)	840,713 (24.4)	731,385 (24.3)	788,929 (24.2)
25–30	905,369 (29.3)	920,298 (29.0)	996,235 (28.9)	840,378 (27.9)	904,578 (27.8)
≥30	104,491 (3.4)	98,471 (3.1)	108,610 (3.2)	96,124 (3.2)	105,678 (3.2)
Hypertension	837,753 (27.1)	845,966 (26.6)	906,313 (26.3)	773,584 (25.7)	832,219 (25.6)
Diabetes	253,083 (8.2)	257,386 (8.1)	275,412 (8.0)	237,054 (7.9)	257,498 (7.9)
Dyslipidemia	479,476 (15.5)	482,558 (15.2)	524,485 (15.2)	444,108 (14.8)	461,546 (14.2)
Current Smoking	764,147 (24.7)	807,490 (25.4)	822,821 (23.9)	736,924 (24.5)	820,845 (25.2)
Alcohol Consumption (yes)	1,407,843 (45.5)	1,511,332 (47.5)	1,624,673 (47.2)	1,433,861 (47.7)	1,601,178 (49.2)
Income Status (<20%)	770,592 (24.9)	717,217 (22.6)	736,450 (21.4)	623,084 (20.7)	629,505 (19.3)

Data are presented as numbers (%); Q, quintile; BMI, body mass index. ^a^ Age- and gender-specific quintiles (see Supplementary Table S1). ^b^ Mean ± standard deviation.

**Table 2:** Incidence rate and adjusted hazard ratio of vitiligo according to height.

Group	Vitiligo Diagnosis	Person-Years	Incidence Rate (Per 1000 Person-Years)	Hazard Ratio (95% CI)	
				Model 1	Model 2
Total					
Q1	4908	26,266,111	0.19	1 (ref.)	1 (ref.)
Q2	5472	27,058,790	0.20	1.09 (1.05, 1.13)	1.11 (1.07, 1.16)
Q3	6516	29,345,746	0.22	1.18 (1.14, 1.23)	1.22 (1.18, 1.27)
Q4	5774	25,634,799	0.23	1.29 (1.16, 1.25)	1.26 (1.21, 1.31)
Q5	6526	27,714,768	0.24	1.27 (1.22, 1.32)	1.36 (1.31, 1.42)
Gender					
Male					
Q1	2150	14,447,218	0.15	1 (ref.)	1 (ref.)
Q2	2455	15,001,287	0.16	1.10 (1.04, 1.17)	1.12 (1.06, 1.19)
Q3	2730	15,390,906	0.18	1.20 (1.13, 1.27)	1.24 (1.17, 1.32)
Q4	2311	13,240,619	0.17	1.18 (1.12, 1.25)	1.24 (1.17, 1.32)
Q5	2750	14,879,483	0.18	1.27 (1.20, 1.34)	1.36 (1.28, 1.45)
Female					
Q1	2758	11,818,893	0.23	1 (ref.)	1 (ref.)
Q2	3017	12,057,502	0.25	1.08 (1.03, 1.14)	1.10 (1.04, 1.16)
Q3	3786	13,954,840	0.27	1.17 (1.11, 1.23)	1.20 (1.14, 1.26)
Q4	3463	12,394,180	0.28	1.21 (1.15, 1.27)	1.26 (1.20, 1.33)
Q5	3776	12,835,285	0.29	1.28 (1.21, 1.34)	1.35 (1.28, 1.42)
Index Age					
Age < 65					
Q1	3792	20,985,874	0.18	1 (ref.)	1 (ref.)
Q2	4220	21,936,313	0.19	1.06 (1.02, 1.11)	1.10 (1.05, 1.15)
Q3	4915	23,617,789	0.21	1.13 (1.09, 1.18)	1.20 (1.15, 1.26)
Q4	4321	20,703,136	0.21	1.14 (1.09, 1.19)	1.23 (1.18, 1.29)
Q5	4861	22,337,748	0.22	1.22 (1.17, 1.27)	1.37 (1.31, 1.44)
Age ≥ 65					
Q1	1116	5,280,236	0.21	1 (ref.)	1 (ref.)
Q2	1252	5,122,476	0.24	1.15 (1.06, 1.24)	1.15 (1.06, 1.25)
Q3	1601	5,727,957	0.28	1.28 (1.19, 1.38)	1.29 (1.20, 1.40)
Q4	1453	4,931,663	0.29	1.34 (1.24, 1.45)	1.36 (1.25, 1.47)
Q5	1665	5,377,020	0.31	1.41 (1.31, 1.52)	1.44 (1.32, 1.56)

Data are presented as numbers; CI, confidence interval; Q1–Q5, Age- and gender-specific quintiles (see Supplementary Table S1). Model 1, adjusted for index age and gender. Model 2, adjusted for index age, gender, BMI, presence of hypertension, presence of DM, presence of hyperlipidemia, smoking, alcohol consumption, and income status. Subgroup analyses were performed by gender and index age.

## Data Availability

Data is contained within the article or Supplementary Material.

## Supplementary Materials

The following are available online at www.mdpi.com/article/10.3390/jcm10173958/s1. Table S1: Table that illustrates cut-off value of the height of quintile according to age and sex.

## References

[1] Bergqvist C., Ezzedine K. (2021). Vitiligo: A focus on pathogenesis and its therapeutic implications. J. Dermatol..

[2] Bergqvist C., Ezzedine K. (2020). Vitiligo: A review. Dermatology.

[3] Rodrigues M., Ezzedine K., Hamzavi I., Pandya A.G., Harris J.E. (2017). New discoveries in the pathogenesis and classification of vitiligo. J. Am. Acad. Dermatol..

[4] Zhang Y., Cai Y., Shi M., Jiang S., Cui S., Wu Y., Gao X.H., Chen H.D. (2016). The prevalence of vitiligo: A meta-analysis. PLoS ONE.

[5] Alikhan A., Felsten L.M., Daly M., Petronic-Rosic V. (2011). Vitiligo: A comprehensive overview Part I. Introduction, epidemiology, quality of life, diagnosis, differential diagnosis, associations, histopathology, etiology, and work-up. J. Am. Acad. Dermatol..

[6] Krüger C., Schallreuter K.U. (2012). A review of the worldwide prevalence of vitiligo in children/adolescents and adults. Int. J. Dermatol..

[7] Grimes P.E., Miller M.M. (2018). Vitiligo: Patient stories, self-esteem, and the psychological burden of disease. Int. J. Women’s Dermatol..

[8] Parsad D., Dogra S., Kanwar A.J. (2003). Quality of life in patients with vitiligo. Health Qual. Life Outcomes.

[9] Ezzedine K., Sheth V., Rodrigues M., Eleftheriadou V., Harris J.E., Hamzavi I.H., Pandya A.G. (2015). Vitiligo is not a cosmetic disease. J. Am. Acad. Dermatol..

[10] Plaza-Rojas L., Guevara-Patiño J.A. (2021). The role of the NKG2D in vitiligo. Front. Immunol..

[11] Roberts G.H.L., Santorico S.A., Spritz R.A. (2020). The genetic architecture of vitiligo. Pigment. Cell Melanoma Res..

[12] Roberts G.H.L., Santorico S.A., Spritz R.A. (2020). Deep genotype imputation captures virtually all heritability of autoimmune vitiligo. Hum. Mol. Genet..

[13] Nath S.K., Majumder P.P., Nordlund J.J. (1994). Genetic epidemiology of vitiligo: Multilocus recessivity cross-validated. Am. J. Hum. Genet..

[14] Kim H.J., Ahn H.S., Kazmi S.Z., Kang T., Kim H.S., Kang M.J., Kim K.B., Kim D.S., Hann H.J. (2021). Familial risk of vitiligo among first-degree relatives and spouses: A population-based cohort study in Korea. J. Investig. Dermatol..

[15] van Geel N., Speeckaert R., Taieb A., Picardo M., Böhm M., Gawkrodger D.J., Schallreuter K., Bennett D.C., van der Veen W., Whitton M. (2011). Koebner’s phenomenon in vitiligo: European position paper. Pigment. Cell Melanoma Res..

[16] Jeon I.K., Park C.J., Lee M.H., Lee D.Y., Kang H.Y., Hann S.K., Choi G.S., Lee H.J., Kim T.H., Lee A.Y. (2014). A multicenter collaborative study by the Korean society of vitiligo about patients’ occupations and the provoking factors of vitiligo. Ann. Dermatol..

[17] Lettre G. (2011). Recent progress in the study of the genetics of height. Hum. Genet..

[18] Silventoinen K., Sammalisto S., Perola M., Boomsma D.I., Cornes B.K., Davis C., Dunkel L., De Lange M., Harris J.R., Hjelmborg J.V. (2003). Heritability of adult body height: A comparative study of twin cohorts in eight countries. Twin Res. Hum. Genet..

[19] Hwang I.C., Bae J.H., Kim J.M., Lee J.M., Nguyen Q.D. (2020). Adult body height and age-related macular degeneration in healthy individuals: A nationwide population-based survey from Korea. PLoS ONE.

[20] Rosenberg M.A., Patton K.K., Sotoodehnia N., Karas M.G., Kizer J.R., Zimetbaum P.J., Chang J.D., Siscovick D., Gottdiener J.S., Kronmal R.A. (2012). The impact of height on the risk of atrial fibrillation: The Cardiovascular Health Study. Eur. Heart J..

[21] Schmidt M., Bøtker H.E., Pedersen L., Sørensen H.T. (2014). Adult height and risk of ischemic heart disease, atrial fibrillation, stroke, venous thromboembolism, and premature death: A population based 36-year follow-up study. Eur. J. Epidemiol..

[22] Lai F.Y., Nath M., Hamby S.E., Thompson J.R., Nelson C.P., Samani N.J. (2018). Adult height and risk of 50 diseases: A combined epidemiological and genetic analysis. BMC Med..

[23] Berliner M.B.-Z., Katz L.H., Derazne E., Levine H., Keinan-Boker L., Benouaich-Amiel A., Gal O., Kanner A.A., Laviv Y., Honig A. (2020). Height as a risk factor in meningioma: A study of 2 million Israeli adolescents. BMC Cancer.

[24] Lee Y.B., Lee J.H., Kang M.J., Kim J.W., Yu D.S., Han K.D., Park Y.G. (2018). Association between height and actinic keratosis: A nationwide population-based study in South Korea. Sci. Rep..

[25] Ribero S., Glass D., Aviv A., Spector T.D., Bataille V. (2015). Height and bone mineral density are associated with naevus count supporting the importance of growth in melanoma susceptibility. PLoS ONE.

[26] Li X., Liang L., Feng Y.-C.A., De Vivo I., Giovannucci E., Tang J.Y., Han J. (2017). Height, height-related SNPs, and risk of non-melanoma skin cancer. Br. J. Cancer.

[27] Di Giovannantonio M., Harris B.H., Zhang P., Kitchen-Smith I., Xiong L., Sahgal N., Stracquadanio G., Wallace M., Blagden S., Lord S. (2021). Heritable genetic variants in key cancer genes link cancer risk with anthropometric traits. J. Med. Genet..

[28] Lee H., Lee M.H., Lee D.Y., Kang H.Y., Kim K.H., Choi G.S., Shin J., Lee H.J., Kim D.H., Kim T.H. (2015). Prevalence of vitiligo and associated comorbidities in Korea. Yonsei Med. J..

[29] Choi C.W., Eun S.H., Choi K.H., Bae J.M. (2017). Increased risk of comorbid rheumatic disorders in vitiligo patients: A nationwide population-based study. J. Dermatol..

[30] Gill L., Zarbo A., Isedeh P., Jacobsen G., Lim H.W., Hamzavi I. (2016). Comorbid autoimmune diseases in patients with vitiligo: A cross-sectional study. J. Am. Acad. Dermatol..

[31] Chen Y.T., Chen Y.J., Hwang C.Y., Lin M.W., Chen T.J., Chen C.C., Chu S.Y., Lee D.D., Chang Y.T., Liu H.N. (2015). Comorbidity profiles in association with vitiligo: A nationwide population-based study in Taiwan. J. Eur. Acad. Dermatol. Venereol..

[32] Teulings H.E., Ceylan E., Overkamp M., Vrijman C., Bos J.D., Nijsten T.E., Wolkerstorfer A., Luiten R.M., van der Veen J.P. (2016). Nonsegmental vitiligo disease duration and female sex are associated with comorbidity and disease extent: A retrospective analysis in 1307 patients aged ≥ 50 years. Br. J. Dermatol..

[33] Narita T., Oiso N., Fukai K., Kabashima K., Kawada A., Suzuki T. (2011). Generalized vitiligo and associated autoimmune diseases in Japanese patients and their families. Allergol. Int..

[34] Lamb M.M., Yin X., Zerbe G.O., Klingensmith G.J., Dabelea D., Fingerlin T.E., Rewers M., Norris J.M. (2009). Height growth velocity, islet autoimmunity and type 1 diabetes development: The diabetes autoimmunity study in the young. Diabetologia.

[35] Gollnick H.P., Bettoli V., Lambert J., Araviiskaia E., Binic I., Dessinioti C., Galadari I., Ganceviciene R., Ilter N., Kaegi M. (2016). A consensus-based practical and daily guide for the treatment of acne patients. J. Eur. Acad. Dermatol. Venereol..

[36] Ohlsson C., Mohan S., Sjögren K., Tivesten A., Isgaard J., Isaksson O., Jansson J.O., Svensson J. (2009). The role of liver-derived insulin-like growth factor-I. Endocr. Rev..

[37] Bach L.A. (2018). What happened to the IGF binding proteins?. Endocrinology.

[38] Seneschal J., Boniface K., D’Arino A., Picardo M. (2021). An update on vitiligo pathogenesis. Pigment. Cell Melanoma Res..

[39] Bach L.A. (2018). 40 years of IGF1: IGF-binding proteins. J. Mol. Endocrinol..

[40] Kuzik N., Myette-Côté É., Carson V., Slater L., Boulé N.G. (2015). Evaluating the effects of metformin use on height in children and adolescents: A meta-analysis of randomized clinical trials. JAMA Pediatr..

[41] Lv Z., Guo Y. (2020). Metformin and its benefits for various diseases. Front. Endocrinol..

[42] Podhorecka M., Ibanez B., Dmoszyńska A. (2017). Metformin–its potential anti-cancer and anti-aging effects. Postep. Hig Med. Dosw..

[43] Lee S., Kim M., Han J.H., Ju H.J., Bae J.M. (2020). P099: An identification of potential therapeutics for vitiligo by mass screening for 1732 medicines in Korean national health insurance database. 프로그램북(구 초록집).

[44] Choi Y.J., Lee D.H., Han K.D., Yoon H., Shin C.M., Park Y.S., Kim N. (2019). Adult height in relation to risk of cancer in a cohort of 22,809,722 Korean adults. Br. J. Cancer.

[45] Hwang Y.S., Oh S.W., Park S.H., Lee J., Yoo J.A., Kwon K., Park S.J., Kim J., Yu E., Cho J.Y. (2019). Melanogenic effects of maclurin are mediated through the activation of cAMP/PKA/CREB and p38 MAPK/CREB signaling pathways. Oxid. Med. Cell Longev..

[46] Lee S.E., Park S.H., Oh S.W., Yoo J.A., Kwon K., Park S.J., Kim J., Lee H.S., Cho J.Y., Lee J. (2018). Beauvericin inhibits melanogenesis by regulating cAMP/PKA/CREB and LXR-α/p38 MAPK-mediated pathways. Sci. Rep..

[47] Sun X., Wang T., Huang B., Ruan G., Xu A. (2020). RIPK1 regulates the survival of human melanocytes upon endoplasmic reticulum stress. Exp. Ther. Med..

[48] Lin X., Meng X., Song Z., Lin J. (2020). Nuclear factor erythroid 2-related factor 2 (Nrf2) as a potential therapeutic target for vitiligo. Arch. Biochem. Biophys..

[49] Zhu S., Long L., Hu Y., Tuo Y., Li Y., Yu Z. (2021). GnRHa/Stanozolol combined therapy maintains normal bone growth in central precocious puberty. Front. Endocrinol..

[50] Kim J.H., Hong A.R., Kim Y.H., Yoo H., Kang S.W., Chang S.E., Song Y. (2020). JNK suppresses melanogenesis by interfering with CREB-regulated transcription coactivator 3-dependent MITF expression. Theranostics.

[51] Fruman D.A., Chiu H., Hopkins B.D., Bagrodia S., Cantley L.C., Abraham R.T. (2017). The PI3K pathway in human disease. Cell.

[52] Zhu L., Lin X., Zhi L., Fang Y., Lin K., Li K., Wu L. (2020). Mesenchymal stem cells promote human melanocytes proliferation and resistance to apoptosis through PTEN pathway in vitiligo. Stem Cell Res. Ther..

[53] Igaz P., Tóth S., Falus A. (2001). Biological and clinical significance of the JAK-STAT pathway; lessons from knockout mice. Inflamm. Res..

[54] Phan K., Phan S., Shumack S., Gupta M. (2020). Repigmentation in vitiligo using janus kinase (JAK) inhibitors with phototherapy: Systematic review and Meta-analysis. J. Dermatolog. Treat..

[55] Tajan M., Pernin-Grandjean J., Beton N., Gennero I., Capilla F., Neel B.G., Araki T., Valet P., Tauber M., Salles J.P. (2018). Noonan syndrome-causing SHP2 mutants impair ERK-dependent chondrocyte differentiation during endochondral bone growth. Hum. Mol. Genet..

[56] Hu M., Chen C., Liu J., Cai L., Shao J., Chen Z., Lin L., Zheng T., Ding X., Li Z. (2020). The melanogenic effects and underlying mechanism of paeoniflorin in human melanocytes and vitiligo mice. Fitoterapia.

[57] Dibble C.C., Cantley L.C. (2015). Regulation of mTORC1 by PI3K signaling. Trends Cell Biol..

[58] Phornphutkul C., Lee M., Voigt C., Wu K.Y., Ehrlich M.G., Gruppuso P.A., Chen Q. (2009). The effect of rapamycin on bone growth in rabbits. J. Orthop. Res..

[59] Cao C., Wan Y. (2009). Parameters of protection against ultraviolet radiation-induced skin cell damage. J. Cell Physiol..

[60] Wan J., Lin F., Zhang W., Xu A., DeGiorgis J., Lu H., Wan Y. (2017). Novel approaches to vitiligo treatment via modulation of mTOR and NF-κB pathways in human skin melanocytes. Int. J. Biol. Sci..

[61] Yu R.X., Hui Y., Li C.R. (2017). Köebner phenomenon induced by striae distensae in a vitiligo patient. Ann. Dermatol..

[62] Iftikhar N., Rahman A., Janjua S.A. (2009). Vitiligo appearing in striae distensae as a Koebner phenomenon. J. Coll. Phys. Surg. Pak..

[63] Feng J., Sang H., Wu F., Liu F., Ni X. (2014). Vitiligo coexistent with striae: Association more than coincidence?. Ann. Dermatol..

[64] Lee J., Lee J.S., Park S.H., Shin S.A., Kim K. (2017). Cohort profile: The national health insurance service-national sample cohort (NHIS-NSC), South Korea. Int. J. Epidemiol..

