# Supplementary material for: Height and Risk of Vitiligo: A Nationwide Cohort Study
Source: J Clin Med. 2021 Aug 31;10(17):3958. doi: 10.3390/jcm10173958 (PMC8432081; doi:10.3390/jcm10173958)
Supplement: Supplementary file 1 [file jcm-10-03958-s001.zip › jcm-1351155-supplementary.pdf]

Supplementary Materials

Supplementary Table S1. Table that illustrates cut-off value of the height of quintile according to age and sex.

Age	Sex	Number of individuals	Height				
			1st quintile	2nd quintile	3rd quintile	4th quintile	Maximum
20–29	Male	1253255	169	172	175	179	209
	Female	1203892	157	160	162	165	198
30–39	Male	2187697	167	171	174	177	207
	Female	932490	155	158	161	164	196
40–49	Male	2039121	165	168	171	174	202
	Female	1851139	153	156	158	161	199
50–59	Male	1581193	163	166	169	172	200
	Female	1687437	151	154	157	160	204
60–69	Male	964084	161	164	167	170	198
	Female	1061340	149	152	154	157	198
≥70	Male	530619	159	162	165	169	198
	Female	688487	145	148	151	154	197
